# Vegetable microbiomes: is there a connection among opportunistic infections, human health and our ‘gut feeling'?

**DOI:** 10.1111/1751-7915.12159

**Published:** 2014-09-03

**Authors:** Gabriele Berg, Armin Erlacher, Kornelia Smalla, Robert Krause

**Affiliations:** 1 Institute of Environmental Biotechnology, Graz University of TechnologyGraz, 8010, Austria; 2 Institute for Epidemiology and Pathogen Diagnostics, Julius Kühn-Institut – Federal Research Centre for Cultivated Plants (JKI)Braunschweig, 38104, Germany; ^3^Section of Infectious Diseases and Tropical Medicine, Department of Internal Medicine, Medical University of GrazGraz 8010, Austria

## Abstract

The highly diverse microbiomes of vegetables are reservoirs for opportunistic and emerging pathogens. In recent years, an increased consumption, larger scale production and more efficient distribution of vegetables together with an increased number of immunocompromised individuals resulted in an enhanced number of documented outbreaks of human infections associated with the consumption of vegetables. Here we discuss the occurrence of potential pathogens in vegetable microbiomes, the impact of farming and processing practices, and plant and human health issues. Based on these results, we discuss the question if vegetables can serve as a source of infection for immunocompromised individuals as well as possible solutions to avoid outbreaks. Moreover, the potentially positive aspects of the vegetables microbiome for the gut microbiota and human health are presented.

## Pathogenicity, (opportunistic) pathogens and immunocompromised individuals

Pathogenicity to humans, animals and plants is the most acclaimed feature of microorganisms. Traditionally, pathogens are defined as causative agents of diseases, guided by Koch's postulates for more than a century and further improved by molecular criteria (Fredericks and Relman, 2014). Next generation sequencing-based technologies have revolutionized our knowledge not only on the microbiome, but also about pathogens drastically (Jansson *et al*., 2012; Berg *et al*., 2009; Bergholz *et al*., 2014). The human microbiome is involved in many more human diseases than recently thought, and microbial imbalances can be responsible for severe diseases (Tremaroli and Bäckhed, 2011; Blaser *et al*., 2013). Pathogen outbreaks are associated with shifts of the whole community including those supporting pathogens as well as opportunistic pathogens (Clemente *et al*., 2012). On the other side, microbial diversity is an important factor determining the invasion of pathogens; reduced diversity supports opportunistic infections (van Elsas *et al*., 2012; Pham and Lawley, 2014).

Opportunistic pathogens usually do not cause disease in a healthy, immunocompetent host; they take advantage of certain situations, for example, from compromised immune system of patients, which presents an ‘opportunity’ for the pathogen to infect. The number of immunocompromised individuals rises continuously worldwide and can be caused not only by recurrent infections, advanced human immunodeficiency virus (HIV) infection and genetic predisposition, but also by medical treatments, for example immunosuppressive agents for organ transplant recipients, chemotherapy for cancer or long-term antibiotic treatments (Klevens *et al*., 2007; Fishman, 2013). A substantial number of opportunistic pathogens cause health-care-associated infections (HAIs) or nosocomial infections, because in health-care settings (e.g. wards, outpatient haemodialysis units, or same-day surgery), the number of immunocompromised individuals is high. In addition, the indoor environments of these settings contain a specific microbiome including diverse opportunistic pathogens (Oberauner *et al*., 2013). HAIs are associated with significant morbidity, mortality and cost. According to the US National Nosocomial Infections Surveillance system, in 2002, the estimated number of HAIs in US hospitals was approximately 1.7 million (Klevens *et al*., 2007). Opportunistic infections remain a major health problem worldwide and can limit immunosuppression therapies (Fishman, 2013). Interestingly, a worldwide study identified a significant association between the risk of death because of opportunistic infections in intensive care units and the global national income (Vincent *et al*., 2014). Although an excellent concordance between US and European definitions of HAIs was reported (Hansen *et al*., 2012), the taxonomic spectrum of opportunistic pathogens varies from hospital to hospital and is influenced by biogeographic aspects. Besides viruses, fungi and protozoa, a long list of bacterial pathogens causes opportunistic infections. The most reported species include the Gram-positive *Staphylococcus aureus* including methicillin-resistant *S. aureus*, *Enterococcus* species (*E. faecalis*, *E. faecium*), and Gram-negative bacteria like *Escherichia coli*, and *Pseudomonas aeruginosa* (Sydnor and Perl, 2011). Moreover, today, the antibiotic-resistant Gram-negative microorganisms, for example *Acinetobacter*, *Enterobacter*, *Klebsiella* (*K. pneumonia, K. oxytoca*), *Proteus*, *Pseudomonas*, *Serratia* and *Stenotrophomonas* are particularly troublesome, especially in the development of hospital-acquired infections (Sydnor and Perl, 2011). HAIs are associated with a broad range of diseases and symptoms: they can cause severe pneumonia, bloodstream infections, urinary tract infections, surgical site infections and other infections. In addition to the direct effects, opportunistic infections and the microbiome may adversely shape the host immune responses (Fishman, 2013).

Patients with cystic fibrosis are specifically prone to opportunistic infections. This hereditary disease affects the epithelial innate immune function in the lung, resulting in exaggerated and ineffective airway inflammation that fails to eradicate pulmonary pathogens. Pulmonary infection is therefore the most challenging problem in the management of cystic fibrosis and is the major determinant of life span and quality of life in affected individuals. Although the most important opportunistic pathogens are again *P. aeruginosa* and *S. aureus*, the number of causative species is higher and also includes the *Burkholderia cepacia* complex, *Burkholderia gladioli*, *Stenotrophomonas maltophilia*, *Achromobacter xylosoxidans*, *Ralstonia*, *Cupriavidus* and *Pandoraea* species (LiPuma, 2010).

Are there common characteristics of opportunistic pathogens? Although opportunistic pathogens have a broad phylogenetic background and include strains affiliated to *Firmicutes* (*Staphylococcus*, *Enterococcus*), *Betaproteobacteria* (*Burkholderia*) and *Gammaproteobacteria* (*Pseudomonas*, *Stenotrophomonas*, *Acinetobacter*, *Klebsiella*, *Escherichia*, *Enterobacter*, *Proteus*, *Serratia*), they share some properties. Opportunistic pathogens occur in natural environments and are often associated with other eukaryotic hosts such as plants. They are often characterized by several of the following properties: (i) r-strategists = copiotrophs, (ii) cultivable, (iii) antagonistic towards other microorganisms, (iv) highly competitive, (v) highly versatile in their nutrition, (vi) hypermutators, (vii) resistant against antibiotics and toxins and (viii) form biofilms. It is important to note that typically these traits were acquired via horizontal gene transfer and are strain specific (Rossi *et al*., 2014). It is predicted that in future decades, other lesser-known pathogens and new bacterial strains of bacteria will emerge as common causal agents of infections (Sydnor and Perl, 2011); therefore, it is important to understand the ecology of potentially emerging pathogens.

## The vegetable microbiome

In a basic study, Leff and Fierer (2013) found that vegetables harboured diverse bacterial communities dominated by the phyla *Actinobacteria*, *Bacteroidetes*, *Firmicutes* and *Proteobacteria*, but their composition was significantly different for each vegetable species. These differences were often attributable to distinctions in the relative abundances of *Enterobacteriaceae* taxa (Leff and Fierer, 2013). This large family of Gram-negative bacteria includes, along with many harmless symbionts, many of the more familiar so-called enteric pathogens that also play an important role as opportunistic pathogens (Brandl, 2006; Rastogi *et al*., 2012). However, according to these studies, they are an important component of the indigenous vegetable microbiome. In addition to raw vegetables, fermented fresh-like vegetables are a substantial part of our diet worldwide, and specific traditional products exist in different areas, for example ‘Kimchi’ in Korea or ‘Sauerkraut’ in Germany. Lactic acid fermentation using indigenous bacteria or starter cultures induce shifts to the bacterial community (Di Cagno *et al*., 2013).

Lettuce has a special position within the vegetable group; it is among the most popular raw-eaten vegetables with a global consumption of 24.6 Mio t (The Statistics Division of the Food and Agriculture Organization of the United Nations) and provides a habitat for specific microbes (Rastogi *et al*., 2012). The authors found high abundances 10^5^–10^6^ colony-forming unit (cfu) g^−1^ fw and diversities with a high proportion of *Enterobacteriaceae* in the phyllosphere of field-grown Romaine lettuce. *Enterobacteriaceae* taxa are present not only in the gammaproteobacterial microbiome of the lettuce phyllosphere und comprise potential beneficial bacteria, but also potential pathogens (Erlacher *et al*., 2014). In the German monitoring system of pathogens, verocytotoxin-producing *Escherichia coli* were found in 1.3% (0.4–3.4) and *E. coli* in 3.8% of the investigated lettuce samples (Käsbohrer *et al*., 2014). Washing steps and adding of detergents to sanitizer solutions failed in decontamination (Keskinen and Annous, 2011). This can be explained by an endophytic colonization of bacteria observed by Berg *et al*. (2014).

**Fig 1:**
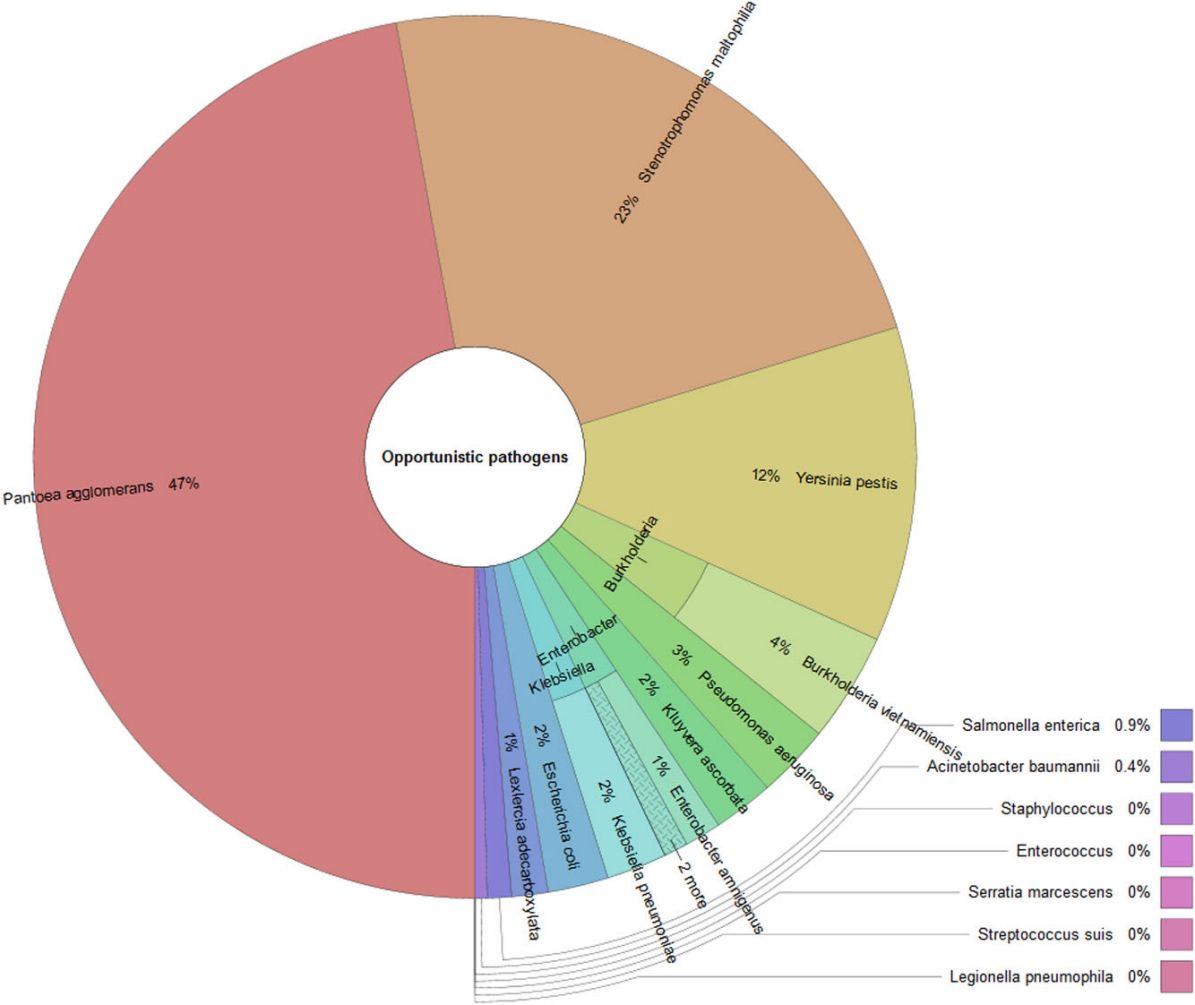
Occurrence and taxonomic structure of opportunistic pathogens in the phyllosphere of *Eruca sativa* Mill. analyzed from a metagenomic data set. The relative abundance is based on the presented taxa and composed of 1.7% of the total bacterial fraction.

**Fig 2:**
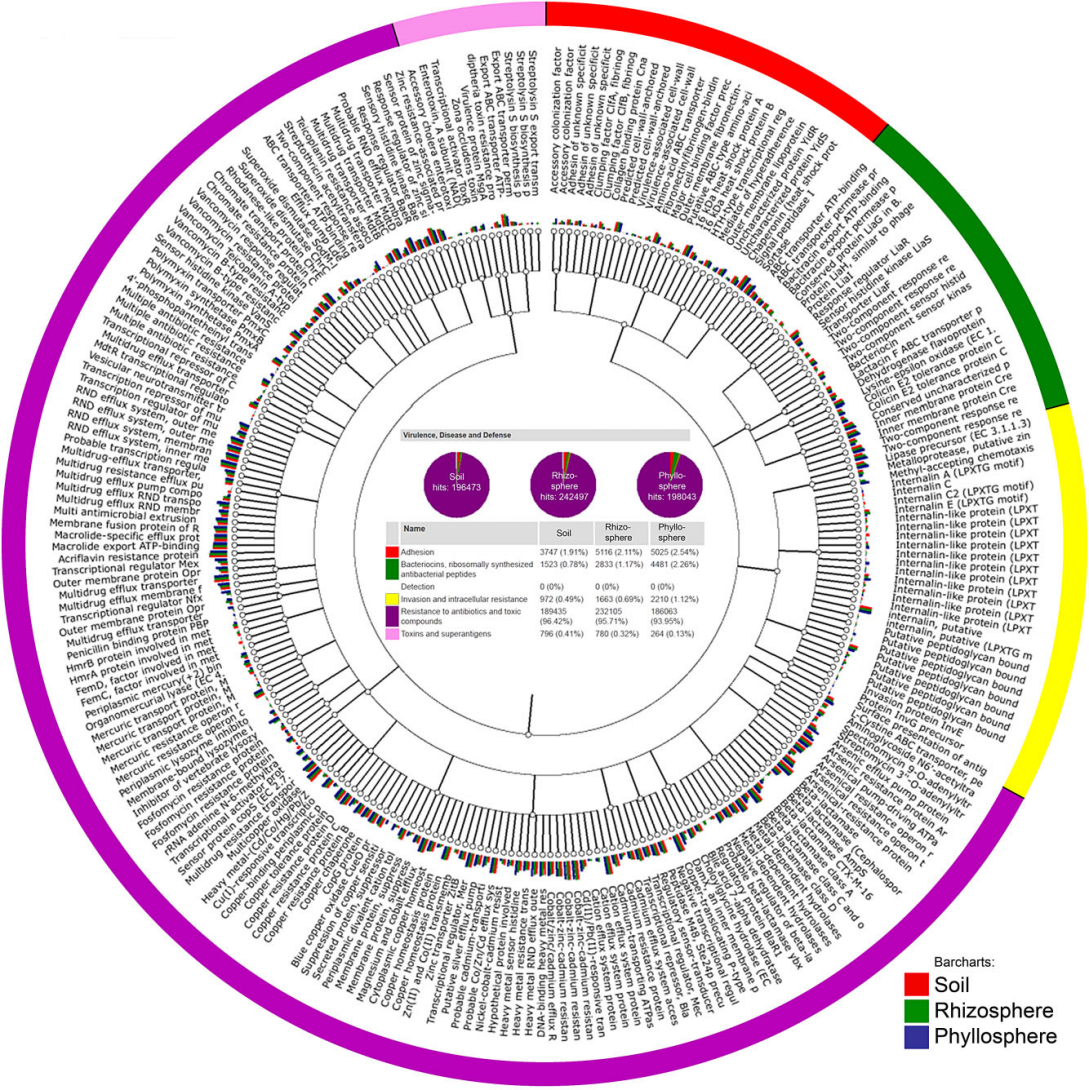
Functional diversity tree of the virulence, disease and defence cluster of *Eruca sativa* Mill. The data were compared with SEED using a maximum e-value of 1e-5, a minimum identity of 60 % and a minimum alignment length of 15 measured in aa for protein and bp for RNA databases. Colour shading indicates classification membership and investigated habitat (bar charts).

Omics approaches are starting to yield practical food safety solutions, but currently, only few studies are available (Bergholz *et al*., 2014). We used our metagenomic dataset of rucola (syn. arugula, *Eruca sativa* Mill.), which is widely popular as a salad vegetable, to detect frequently reported opportunistic pathogens (A. Erlacher and G. Berg, unpubl. data). Altogether, using the Greengenes database, the fraction of opportunistic pathogens comprised about 1.7% of the total bacterial community with the dominance of *Pantoea agglomerans* and *Stenotrophomonas maltophilia* – both are known for their ambivalent interactions with plants and humans (Fig. 1). In addition, a high proportion of genes involved in functions such as virulence, disease and defence were identified in the rucola phyllosphere, rhizosphere and the surrounding bulk soil (Fig. 2). This cluster contains functions for the subgroups responsible for adhesion, bacteriocin production and ribosomally synthesized antibacterial peptides, detection, invasion and intracellular resistance, resistance to antibiotics and toxic compounds, and toxins and superantigens. Interestingly, except the subgroup of toxins and superantigens, which is absent in the phyllosphere, comparable patterns for all three investigated habitats were found.

Farming and processing practices have an important influence on the composition of associated microbial communities (Leff and Fierer, 2013). Larger scale production and more efficient distribution of fresh vegetables over the past two decades have contributed to an increase in the number of illness outbreaks (Olaimat and Holley, 2013). Organic farming practices can differ from conventional farming practices, including the types of fertilizer and pesticides that are used, and these differences have the potential to impact microbial community structure associated with vegetables; they are often characterized by a higher microbial diversity (Schmid *et al*., 2011; Leff and Fierer, 2013). During the last decades, the usage of antibiotics in animal husbandry has promoted the development and abundance of antibiotic resistance in farm environments drastically (Woolhouse and Farrar, 2014). Especially, manure is a reservoir of resistant bacteria and antibiotic compounds, and its application to agricultural soils is assumed to significantly increase antibiotic resistance genes and selection of resistant bacterial populations in soil (Heuer *et al*., 2011; Jechalke *et al*., 2014). From the rhizosphere, these populations can invade into the endosphere of plants and here enter the food chain of humans. Pathogen contamination of fresh products may originate before or after harvest, but once contaminated, products are difficult to sanitize (Olaimat and Holley, 2013). However, food-processing practices also have an important impact on the structure of the vegetable microbiome and food safety (Olaimat and Holley, 2013). For example, intermediate disturbances (e.g. by minor biotic or abiotic stresses) can enhance the relative abundance of *Enterobacteriaceae* (A. Erlacher and G. Berg, unpubl. data). Although outbreaks of enteric pathogens associated with fresh produce in the form of raw or minimally processed vegetables and fruits have recently increased, the ecology of enteric pathogens outside of their human and animal hosts is less understood (van Overbeek *et al*., 2014). The relatively infrequent outbreaks associated with pre-harvest contamination with *Shigella*, an organism with humans as its major reservoir, and the relative high frequency of those associated with *Salmonella* or *Shiga*-toxin-producing *Escherichia coli*, organisms with animals as their major reservoirs, underline the role of domestic and wild animals as dominant sources of pre-harvest contamination of vegetables like salads (Allerberger and Sessitsch, 2009).

## Opportunistic pathogens in the vegetable microbiome

**Fig 3:**
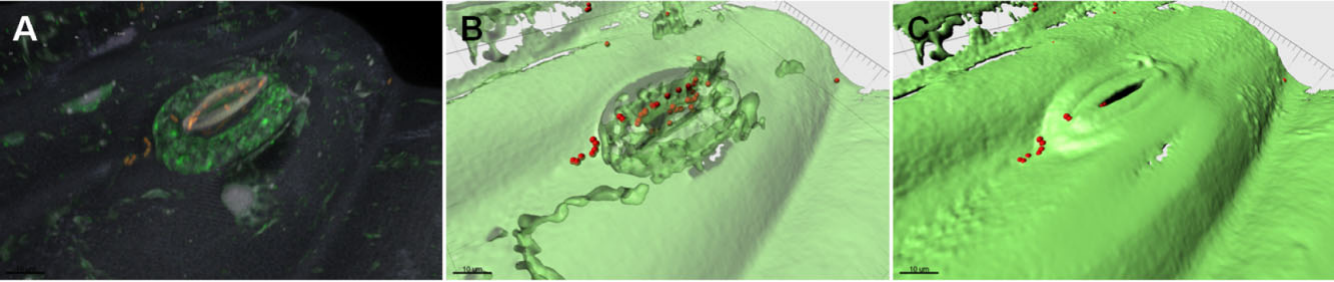
*Escherichia coli* cells on lettuce leaves and colonization of stomata visualized by Fluorescence *in situ* hybridization coupled with confocal laser scanning microscopy.A. Rendering of a confocal Z-stack volume.B and C. Isosurface models of A showing bacteria inside the stoma.

Plants, especially their endospheres and rhizospheres are important reservoirs for emerging opportunistic pathogens (Berg *et al*., 2005; Mendes *et al*., 2013). The number of documented outbreaks of human infections associated with the consumption of raw vegetables has increased in recent years (Buck *et al*., 2003). Diverse human pathogens are able to colonize vegetables including *E. coli* pathovars (Buck *et al*., 2003; van Overbeek *et al*., 2014). Figure 3 shows the invasion of *E. coli* cells into lettuce leaves via stomata after bacterial treatment. There are many plant-associated genera, including *Burkholderia*, *Enterobacter*, *Pseudomonas*, *Ralstonia*, *Serratia*, *Staphylococcus* and *Stenotrophomonas* that enter bivalent interactions with plant and human hosts. Several members of these genera show plant growth promoting as well as excellent antagonistic properties against plant pathogens; therefore, they are utilized to control pathogens to promote plant growth (Berg *et al*., 2005). However, many strains also successfully colonize human organs and tissues and thus cause diseases. One reason is that similar or often identical factors allow recognition, adherence and invasion of plant and human hosts (Berg *et al*., 2005). Well-studied examples of this group are the Gram-negative, often multi-resistant species *Pseudomonas aeruginosa* and *Stenotrophomonas maltophilia*. Both were found as abundant members of plant microbiomes, and strains belonging to these species are characterized by a high versatility at genotypic and phenotypic level. Surprisingly, the pan-genome of *P. aeruginosa* has a larger genetic repertoire than the human genome, which explains the broad metabolic capabilities of *P. aeruginosa* and its ubiquitous distribution in habitats (Tümmler *et al*., 2014). Moreover, the popular plant model, *Arabidopsis thaliana*, has been used to successfully identify novel *P. aeruginosa* genes that are involved in virulence (Baldini *et al*., 2014). *Stenotrophomonas maltophilia* strains show a similar degree of diversity (Berg *et al*., 1999; Ryan *et al*., 2009; Alavi *et al*., 2014). Here, polymorphic mutation frequencies of clinical and environmental *S. maltophilia* populations explain the adaptation to new niches (Turrientes *et al*., 2010). Plant-associated populations have a broader diversity, and only those with a high mutation frequency (hypermutators) were able to adapt to clinical environments and human hosts. Although *S. maltophilia* strains cause a high number of nosocomial infections, only unspecific virulence factors, for example proteases and siderophores, were identified (Ryan *et al*., 2009). Strains belonging to this species persist and display multi-resistance; only a reduced indigenous microbiome gives an opportunity for the pathogen to infect humans. In natural habitats, *S. maltophilia* strains colonize dicotyledonous plants, which produce diverse secondary, antimicrobial metabolites, for example medicinal plants, eucalyptus and *Brassicaceae* (Ryan *et al*., 2009). To survive in such plant habitats, efflux pumps are used, which are also responsible for their multi-resistance against clinically used antibiotics (García-León *et al*., 2014). However, studies show a high plasticity as well as specificity of genomes and epigenomes at strain level, which can contribute to the development of virulent strains (Alavi *et al*., 2014).

## The role of potential pathogens for plants and humans

The plant microbiome plays an important role for plant growth and health and depends on factors such as the plant species, the cultivar and the soil type (Berg and Smalla, 2009; Berg *et al*., 2009; Schreiter *et al*., 2014). Microorganisms can support the nutrient uptake and produce a broad range of phytohormons or influence the latter. Another important function is the involvement of plant-associated bacteria in pathogen defence (Mendes *et al*., 2013). Many pathogens attack plants, especially fungi, oomycetes and nematodes; it is estimated that they cause more than one third of yield losses worldwide. Whereas resistance against leaf pathogens is often encoded in the plant genome, it is difficult to find resistance genes against soil-borne pathogens. Cook and colleagues suggested already in 1995 that antagonistic rhizobacteria fulfil this function – this group acts also as human opportunistic pathogens. Besides direct antagonism, plant-associated bacteria can induce a systemic response in the plant, resulting in the activation of plant defence mechanisms (Pieterse *et al*., 2003).

Another hypothesis is that the plant microbiome has also a positive function for human health by stimulating our immune system and enhancing microbial diversity in the gut microbiome. Recently, Hanski and colleagues (2012) showed a correlation between bacterial diversity and atopy as shown through significant interactions with *Enterobacteriaceae*. Furthermore, they showed a positive association between the abundance of *Acinetobacter* and interleukin-10 expression in peripheral blood mononuclear cells in healthy human individuals. Interleukin-10 is an anti-inflammatory cytokine and plays a central role in maintaining immunologic tolerance to harmless substances (Lloyd and Hawrylowicz, 2009). Endotoxin derived from Gram-negative bacteria, such as *Enterobacteriaceae*, is known to have allergy-protective and immuno-modulatory potential (Doreswamy and Peden, 2011). If plants are a natural reservoir of *Enterobacteriaceae*, then these bacteria must have been a ‘natural’ part of our diet for a long time. Taking into account how many vegetables and fruits are eaten by people worldwide, these outbreaks seem to be more of an accident than the norm, particularly considering that traditionally, food was not processed and sterilized before eating. Therefore, the function of the plant-associated microbiome as an immune-stimulant or ‘natural vaccination’ was suggested by Berg and colleagues (2014). Interestingly, there is an overlap between the plant and human gut microbiome with respect to species composition and function (Ramírez-Puebla *et al*., 2013). Recent studies showed that the stomach does not pose a strict barrier for microbial passage as was previously thought; it is colonized by a broad diversity of species (von Rosenvinge *et al*., 2013). David and colleagues (2014) also recently provided additional evidence for the survival of food-borne microbes (both animal- and plant-based diet) after transit through the digestive system, and that food-borne strains may have been metabolically active in the gut. Microbial diversity in our gut ecosystem has an enormous impact on the host and *vice versa* connected by gut–brain crosstalk, which was revealed as complex, bidirectional communication system (Mayer, 2011). Interesting relationships were detected recently, for example between the gut microbiome and the development of obesity, between cardiovascular disease and metabolic syndromes (Tremaroli and Bäckhed, 2011) and also between motivation and higher-cognitive functions, including intuitive decision-making (Mayer, 2011). This important relationship is confirmed by the enormous success of faecal transplantations (De Vrieze, 2013). The impact of the vegetable microbiome on our health seems to be important and needs more attention in the future.

## Solutions and conclusions

The gathered data indicate that the interplay of different microbiomes is very important. The microbiomes of vegetables, humans as well as in built environment such as hospitals seems to be well connected (Ramírez-Puebla *et al*., 2013; Berg *et al*., 2014). Microbial diversity is an important issue to avoid pathogen outbreaks, which can be often explained by microbial imbalances and poorness (van Elsas *et al*., 2012; Pham and Lawley, 2014). Therefore, to maintain and support microbial diversity is of interest to stabilize ecosystems. Here also, biotechnological solutions are already shown successfully for agriculture (Berg *et al*., 2009) or human health (Petrof and Khoruts, 2014). Probiotics, prebiotics, and synbiotics for plants as well as humans can provide support of the indigenous microbiome (De Vrese and Schrezenmeir, 2008). However, human activities contribute to fast changes of farming and processing practices of vegetables and also influence the structure and function of vegetable-associated bacteria. By horizontal gene transfer multi-resistant super-bugs can develop – a scenario that should be avoided by a careful assessment of new techniques and processes. The new methods and omics technologies in microbial ecology allow these evaluations in great depth and can hopefully contribute to new environmentally friendly solutions. Moreover, to integrate epigenetics in multi-omics techniques opens existing opportunities for new discoveries (Chen *et al*., 2014).

The following points can be concluded:

- Vegetable microbiomes are highly diverse; the composition of species varies for different vegetable species and is strongly influenced by biogeographic aspects and farming and food processing practices. *Enterobacteriaceae* belong to the indigenous microbiota and are key stone species.
- The vegetable microbiome is a reservoir for a long list of opportunistic and emerging pathogens. It is predicted that in future decades, other lesser-known pathogens and new strains of bacteria will emerge as common causes of infections.
- Opportunistic pathogens have a broad phylogenetic background (e.g. *Firmicutes*, *Beta*- and *Gammaproteobacteria*) and occur in natural environments or associated with eukaryotic hosts.
- Many potentially opportunistic pathogens have an endophytic lifestyle. This shows not only their intimate interactions with their host, but also results in difficulties of decontamination.
- In immunocompetent hosts, these bacteria can stimulate the immunosystem and enhance microbial diversity to maintain our health. Moreover, they can contribute to the diversity of our gut microbiome. This diversity is important not only to avoid the development of diseases such as obesity, cardiovascular disease and metabolic syndromes, but also for our motivation and higher-cognitive functions, including intuitive decision-making.
- In immunocompromised individuals, opportunistic pathogens can cause severe infections. These infections include HAIs like pneumonia, bloodstream infections, urinary tract infections, surgical site infections and also diarrhoea.
- To understand the structure and function of microbiomes and their interplay is important to manipulate, reduce or maintain microbial diversity for human and ecosystem health. While multi-omics integration offers technical solutions, probiotics, prebiotics, and synbiotics can provide biotechnological solutions.
